# Inner and Outer Portions of Colonic Circular Muscle: Ultrastructural and Immunohistochemical Changes in Rat Chronically Treated with Otilonium Bromide

**DOI:** 10.1371/journal.pone.0103237

**Published:** 2014-08-14

**Authors:** Chiara Traini, Maria Simonetta Faussone-Pellegrini, Stefano Evangelista, Katia Mazzaferro, Gianluca Cipriani, Paolo Santicioli, Maria Giuliana Vannucchi

**Affiliations:** 1 Dept of Experimental and Clinical Medicine, Histology and Embryology Research Unit, Florence, Italy; 2 Menarini Ricerche SpA, Preclinical Development, Florence, Italy; 3 Enteric Neuroscience Program, Division of Gastroenterology, Dept of Physiology and Biomedical Engineering, Mayo Clinic, Rochester, MN, United States of America; National Institute of Agronomic Research, France

## Abstract

Rat colonic circular muscle, main target of otilonium bromide (OB) spasmolytic activity, is subdivided in an inner and outer portion. Since the inner one is particularly rich in organelles involved in calcium availability (caveolae, smooth endoplasmic reticulum, mitochondria), the expression of specific markers (Caveolin-1, eNOS, calreticulin, calsequestrin) in comparison with the outer portion was investigated. The possible changes of these organelles and related markers, and of muscarinic receptors (Mr2) were then studied after OB chronic exposition. Rats were treated with 2–20 mg/kg/OB for 10 or 30 days. Proximal colon was processed by electron microscopy, immunohistochemistry, and western blot. In colon strips the stimulated contractility response to muscarinic agonist was investigated. The inner portion showed a higher expression of Caveolin-1 and Mr2, but not of eNOS, calreticulin and calsequestrin, compared to the outer portion. Chronic OB treatment caused similar ultrastructural and immunohistochemical changes in both portions. Organelles and some related markers were increased at 10 days; Mr2 expression and muscle contractility induced by methacholine was increased at 30 days. The present findings: 1) provide new information on the immunohistochemical properties of the inner portion of the circular layer that are in favour of a role it might play in colonic motility distinct from that of the outer portion; 2) demonstrate that chronically administered OB interferes with cell structures and molecules responsible for calcium handling and storage, and modifies cholinergic transmission. In conclusion, chronic OB administration in the colonic circular muscle layer directly interacts with the organelles and molecules calcium-related and with the Mr2.

## Introduction

Morphological studies in the human, mouse and rat colonic muscle coat [1]–[3] showed that the circular muscle layer (CM) can be distinct in two portions: an outer and thicker one made of smooth muscle cells (SMC) with typical features, and an inner one made of a few rows of SMC particularly rich in smooth endoplasmic reticulum (SER), caveolae and cell-to-cell junctions with the neighbour pacemaker cells, i.e. the interstitial cells of Cajal (ICC). All these features might be associated with a peculiar role of this layer in colonic contractility. To date, no information is available on qualitative or quantitative differences in specific markers between these two layers either in physiological or pathological conditions.

Otilonium bromide (OB) is a quaternary ammonium derivative used for the treatment of gut motility disorders such as the irritable bowel syndrome (IBS) [4]–[6]. In humans, orally administered OB ameliorates IBS symptoms [7], [8] and, by a double-blind placebo-controlled clinical trial, a beneficial long-lasting effect present also after its interruption has been recently reported [9].

*In vitro* studies showed that OB behaves as NK2 receptor (NK2r) and muscarinic receptor type-2 (Mr2) antagonist and blocks L- and T-type Ca^2+^ channels [10], [11]. We recently demonstrated in the rat colon that chronic OB treatment, at doses and administration time comparable with those utilized in humans, caused a significant reduction of substance P (SP) in the myenteric neurons, an increase of the muscular variant of neuronal nitric oxide synthase (nNOSα), a redistribution of the L-type Ca^2+^ channels and hypersensitivity of NK1r in the SMC of the circular layer [12]. These findings were interpreted as the consequence of the interaction between the drug and L-type Ca^2+^ channels. Further, it was reported that 30 days of OB treatment caused in the rat colon a significant decrease in the neuronal variant of NOS (nNOSβ) in the myenteric plexus associated with an up-regulation of excitatory neurotransmission in the CM. It was concluded that chronic OB treatment modifies the balance between inhibitory and excitatory neurotransmission [13]. All these findings indicate that OB owns a broader spectrum of actions than expected by the *in vitro* results. In summary, chronic administration of OB indicates that: i) the colonic CM seems to be the main target of OB actions; ii) most OB actions are likely involved in the storage and handling of calcium; and iii) there is an increase in excitatory neurotransmission. Kinetic experiments have shown that OB is poorly systemically absorbed and accumulates in the muscle wall of the large intestine where it exerts its spasmolytic activity [14], [15].

In the present study we investigated the colonic CM of rats chronically treated with OB, with particular regard to the inner portion of this layer. At first, the expression of markers related to the organelles involved in calcium availability, such as caveolae, SER and mitochondria, was investigated and compared to that of the outer portion. Second, by transmission electron microscopy (TEM), we ascertained changes in the features of these organelles; by immunohistochemistry and western blot, changes in the expression of markers related to the above organelles and of muscarinic receptors; by functional experiments, the possible involvement of the cholinergic system in the increased excitatory neurotransmission.

## Materials and Methods

### Animals

Male Wistar rats (n = 42; Harlan Laboratories, Udine, Italy) weighing 199±2 g were housed two per cage under standardized temperature and humidity, kept on a 12 h light/dark cycle with free access to food and water. The animals were randomly divided into 5 groups (n = 6–9 animals/group): control group and OB treated rats. In the treated groups, the drug (2 or 20 mg/kg/day), that is very soluble in water, was added in drinking water for 10 or 30 days (times chosen on the basis of OB standard treatment in IBS [15]) and its concentration was adjusted every 2 days according to the change in body weight and water intake, as previously described [12]. The control group received only water. At the end of treatment the animals were sacrificed by CO_2_ inhalation, the abdomen was opened and samples of the proximal colon (3–5 cm) quickly removed from 1 cm below the ileocecal junction. Four tissue samples from the colon of each rat were taken and randomly assigned to the different procedures: immunohistochemistry, electron microscopy, western blot analysis and functional studies. All animal procedures were conducted according to the Italian Guidelines for Animal Care (DL 116/92) and the European Communities Council Directive (86/609/EEC). The study was approved by Menarini Ricerche committee for animal experimentation and by Italian Minister of Health.

### Immunohistochemistry

Full thickness samples of the proximal colon, 1 cm in length, were randomly taken (10 control rats, 5 treated rats/each dose for each experimental set) and immediately fixed in 4% paraformaldehyde in 0.1 M phosphate buffered saline (PBS) pH 7.4, for 4–6 h at 4°C. Then they were placed in 30% sucrose in PBS, at 4°C overnight (ON), embedded in a killik cryostat medium compound (Bio-Optica, Milan, Italy) and frozen at −80°C. Transverse sections 8 µm thick were cut with a cryostat and collected on polylysine coated slides. These sections were then pre-incubated in 1.5% bovine serum albumin (BSA) in PBS, pH 7.4 with 0.5% Triton X-100, for 20 min at room temperature (RT) to minimize non-specific binding. The sections were incubated with the primary antibodies (Table 1) diluted in 0.5% Triton and 1.5% BSA in PBS ON at 4°C. The following day, the sections were washed in PBS and then incubated for 2 h at RT in the presence of Alexa Fluor 48- or 568-conjugated secondary antibodies (Table 2) diluted 1∶333 in 0.5% Triton and 1.5% BSA in PBS. At the end of incubation, the sections were washed in PBS and mounted in an aqueous medium (Fluoremount, Sigma, Milan, Italy). Double labelling was performed as follows: after the first incubation as described above, the sections were incubated again with another primary antibody and with its specific secondary antibody. Negative controls were simultaneously performed omitting the primary antibody to exclude the presence of non-specific immunofluorescence staining. The immunoreaction products were observed under an epifluorescence Zeiss Axioskop microscope (Mannheim, Germany) and were acquired using an AxioCam, HRm digital camera (Zeiss, Mannheim, Germany). The fluorescent signal at Axioskop was obtained using a 488- or 568-nm excitation wavelength for green or red immunofluorescence, respectively.

**Table 1:** List of primary antibodies.

Primary antibodies	Host	Characteristics	Dilution IHC-WB	Producer
Anti-Cav-1	Mouse	Monoclonal antibody against human caveolin (aa residues 1–178). Code: 610406	1∶250 1∶2000	BD Transduction Labs, Lexington, KY, USA
Anti-eNOS	Rabbit	Polyclonal antibody against bovin eNOS (aa residues 598–612). Code: ALX-210-505/1	1∶200 1∶1000	Alexis, San Diego, CA, USA
Anti-eNOS	Rabbit	Polyclonal antibody against bovin eNOS (aa residues 599–613). Code: 482726	1∶200 1∶1000	Chemicon, Temecula, CA, USA
Anti-Calreticulin	Chicken	Polyclonal antibody against calreticulin (aa residues 24–43). Code: PA1-902A	1∶200 1∶1000	Thermo Scientific, Runcorn, UK
Anti-Calsequestrin	Mouse	Monoclonal antibody against rabbit skeletal muscle sarcoplasmatic reticulum. Code: ab2824	1∶200 1∶1000	Abcam, Cambridge, UK
Anti-c-kit	Rabbit	Polyclonal antibody against human c-kit (aa residues 963–976). Code: A4502	1∶300	Dako, Glostrup, Denmark
Anti-αSMA	Mouse	Monoclonal antibody against N-terminal synthetic decapeptide of αSMA. Code: A-2547	1∶1000	Sigma Chemical Company, St.Louis, MO, USA
Anti-ChAT	Goat	Polyclonal antibody against human placental ChAT. Code: AB144P	1∶200 1∶1000	Chemicon,Temecula, CA, USA
Anti-Mr2	Rabbit	Polyclonal antibody against human M2R (aa residues 225–356). Code: AMR-002	1∶50	Alomone Labs, Jerusalem, Israel
Anti-Mr2	Rat	Monoclonal antibody against M2R (aa residues 225–359). Code: MAB367	1∶400	Chemicon, Temecula, CA, USA
Anti-βactin	Rabbit	Polyclonal antibody against C-terminal of β-actin. Code: A-2066	1∶20000	Sigma Chemical Company, St.Louis, MO, USA

**Table 2:** List of secondary antibodies.

Secondary antibodies	Host	Characteristics	Dilution IHC	Producer
Anti-Mouse	Goat	Conjugated anti-IgG (Fab fragment)	1∶333	Invitrogen, San Diego, CA, USA
Anti-Rabbit	Goat	Conjugated anti-IgG (Fab fragment)	1∶333	Jackson ImmunoResearch West Grove, PA, USA
Anti-Chicken	Goat	Conjugated anti-IgG (Fab fragment)	1∶333	Invitrogen, San Diego, CA, USA
Anti-Goat	Donkey	Conjugated anti-IgG (Fab fragment)	1∶333	Invitrogen, San Diego, CA, USA
**Secondary antibodies**	**Host**	**Characteristics**	**Dilution WB**	**Producer**
Anti-Mouse	Goat	Conjugated anti-IgG (H+L)	1∶15 000	Jackson ImmunoResearch
Anti-Rabbit	Goat	Conjugated anti-IgG (H+L)	1∶15 000	Jackson ImmunoResearch
Anti-Chicken	Rabbit	Conjugated anti-IgG (H+L)	1∶15 000	Jackson ImmunoResearch
Anti-Goat	Rabbit	Conjugated anti-IgG (H+L)	1∶15 000	Jackson ImmunoResearch
Anti-Rat	Rabbit	Conjugated anti-IgG (H+L)	1∶15 000	Jackson ImmunoResearch

### Electron microscopy

Tissue was collected from the ascending colon of 5 animals from each group (controls and OB treated rats/each dose for each experimental set). We used colonic strips, 1 mm×3 mm long. These strips were immediately cut after the excision and fixed for 6 h in a solution of 2% glutaraldehyde 0.1 M in cacodylate buffer (pH 7.4). After four rinses in the cacodylate-buffered solution containing 0.22 M sucrose, the strips were post-fixed for 1 h in 1% OsO_4_ in 0.1 M PBS. Dehydration was carried out in graded ethanol and the strips were then embedded in Epon using flat moulds to obtain sections with the circular muscle cut in cross-section. Semi-thin sections, obtained with a LKB-NOVA ultramicrotome (Stockholm, Sweden), were stained with a solution of toluidine blue in 0.1 M borate buffer and then observed under a light microscope to select areas away from the strip edges and with no apparent signs of mal-fixation or processing artefacts. Ultra-thin sections of these selected areas were obtained with the LKB NOVA ultramicrotome using a diamond knife and stained with a saturated solution of uranyl acetate in methanol (50∶50) per 12 min at 45°C, followed by an aqueous solution of concentrated bismuth subnitrate per 10 min at RT. At least 10–20 ultra-thin sections from all four strips of each animal were examined under a JEOL 1010 electron microscope (Tokyo, Japan) and photographed.

### Western blot

Full-thickness samples of the proximal colon (10 control rats, 5 treated rats/each dose for each experimental set) were quickly minced and homogenized with a tissue homogenizer (Ing. Terzano, Milan, Italy) in a cold lysis buffer composed of: 10 mM Tris/HCl pH 7.4; 10 mM NaCl; 1.5 mM MgCl_2_; 2 mM Na_2_EDTA; 1 mM phenylmethylsulfonyl fluoride (PMSF); 1% Triton X-100 added with 1X Sigmafast Protease Inhibitor cocktail tables (Sigma-Aldrich, Milan, Italy). Upon centrifugation at 13000 g for 30 min at 4°C, the supernatants were collected, and the total protein content was measured spectrophotometrically using a micro BCA Protein Assay Reagent Kit (Pierce, IL, USA). Samples (70 µg of proteins per well) and appropriate molecular-weight markers (Bio-Rad, Hercules, CA) were loaded onto a 7.6% or 10% SDS-PAGE gel and resolved by standard electrophoresis. The proteins were then blotted (150 V, 1 h) onto nitrocellulose membranes (Amersham Biosciences, Cologno Monzese, Italy). After thorough washings in PBS containing 0.1% Tween-20 (PBS-T, Sigma- Aldrich), the membranes were treated with blocking buffer made by 5% non-fat dry milk (Sigma-Aldrich) diluted in PBS-T for 1 h at RT. Then they were incubated with the primary antibodies ON at 4°C while being stirred (Table 1). The immune reaction products were revealed by incubating membranes with appropriated peroxidase-conjugated secondary antibodies (Table 2) for 1 h at RT. Immunoreactivity (IR) was detected by an ECL chemiluminescence reagent (Immune-Star HRP Chemiluminescent Kit, Bio Rad, Hercules, CA, USA). In order to normalize the values of each antibody, all western blot runs were stripped (Stripping buffer, Thermo scientific, Rockford, IL, USA) and then immunostained with anti-βactin (Table 1), assuming actin as the control housekeeping protein. The western blot immunoreactive bands were revealed using ImageQuant 350 Imager (GE Healthcare, Buckinghamshire, UK).

### Functional studies

Segments from proximal colon were transferred into an oxygenated (95% O_2_ and 5% CO_2_) Krebs solution of the following composition (mM): NaCl 119; NaHCO_3_ 25; KH_2_PO_4_ 1.2; MgSO_4_ 1.5; KCl 4.7; CaCl_2_ 2.5 and glucose 11. Afterward, they were opened along the mesenteric border and pinned to a Sylgard base with the mucosa facing upwards. The mucosal layer was removed by gentle scraping and two muscle strips (3 mm wide by 10 mm long) from each segment were cut in the direction of circular muscle cells.

The strips were placed in a 5-ml organ bath filled with oxygenated Krebs solution at 37°C and connected to isometric force transducers (Ugo Basile, VA, Italy) under an initial tension of 10 mN. Mechanical activity was digitally recorded by an Octal Bridge Amplifier connected to a PowerLab/8sp hardware system (AD Instruments, Bella Vista, MSW, Australia). After a stabilization period of 60 min, the spontaneous mechanical activity was recorded for 15 min and then each preparation was exposed to a single maximal contractile concentration (1 µM) of the Mr2 receptor agonist methacholine (Sigma-Aldrich, Saint Louis, USA). All salts used were of analytical grade and purchased from Merck (Darmstadt, Germany).

### Quantitative and statistical analysis

Quantification of caveolae and SER cisternae was done in micrographs (5 per each group of rats) taken at the same enlargement (x30.000) and containing at least two SMC profiles. Cell perimeters were measured (µm) and the number of caveolae was expressed as mean ± s.e.m *per* µm. The number of SER cisternae was expressed as mean ± s.e.m *per* cell profile. The length of the SER cisternae was also measured and reported as maximal length and ranging values.

Quantitative analysis of the Cav-1- and eNOS-IR structures was performed in transverse sections from control and treated specimens. Digitized images of the muscle wall (4 sections per animal; 3 animals per group, for a total of 12 sections for each antibody) were acquired using a 40x objective. Each photograph was analysed using ImageJ (NIH, Bethesda, USA) to evaluate the intensity of labelling and quantify the area of IR structures. The photographs’ threshold values were set in order to analyse the structures of interest exclusively. Care was taken to maintain the same threshold in the photographs from the control and OB-treated animals. For the analysis of the area, the labelling was converted to a binary image, the number of pixels above threshold was counted and the percentage area containing IR structures was calculated. The results are expressed as IR pixels ± s.e.m.

The quantitative analysis of WB IR bands was performed by computer-assisted densitometry with each band corresponding to a single specimen by using the QuantityOne analysis software (Bio-Rad, Hercules, CA, USA).

Methacholine induced contractile activity was valuated by means of Chart 4.2 software (AD Instruments). All the results were analyzed using Graph Pad Prism 5.0 (GraphPad Software, Inc. San Diego, CA, USA).

Differences between multiple groups of value were examined by the One-Way ANOVA, followed by the Newman-Keuls multiple comparison test or unpaired student T test, as appropriate. A value of p<0.05 was considered statistically significant.

## Results

### Transmission electron microscopy (TEM)

#### Controls

**Figure 1:**
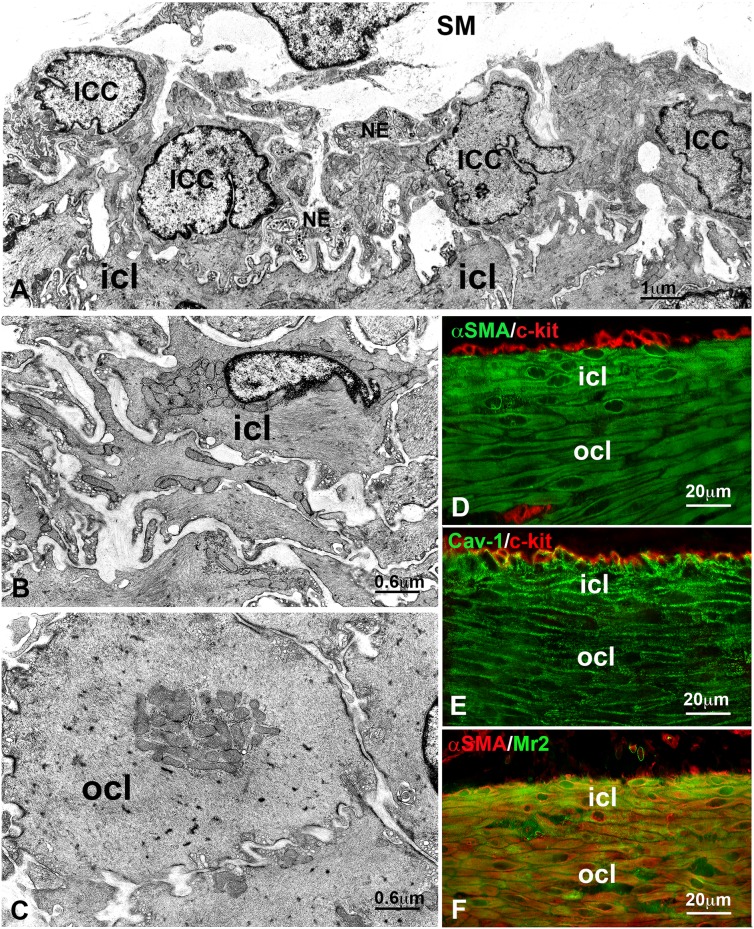
Transmission electron microscopy (TEM) and immunohistochemistry (IHC) of the circular muscle layers in controls. **A–C**. TEM. **A**: An almost continuous monolayer of interstitial cells of Cajal (ICC) covers the innermost portion (icl) of the circular muscle. On the upper side is the submucosa (SM). Several nerve endings (NE) are close to both the ICC and the icl smooth muscle cells. Bar = 1 µm. **B**: Detail of the peculiar morphology of the smooth muscle cells of the icl. Bar = 0.6 µm. **C**: Detail of the typical morphology of the smooth muscle cells of the outer portion of the circular muscle layer (ocl). Bar = 0.6 µm. **D–F**: IHC. **D**: double labelling with αSMA (green) and c-kit (red). The icl is more intensely labelled with αSMA than the ocl. The ICC are c-kit-positive (red) and αSMA-negative. **E**: double labelling with Cav-1 (green) and c-kit (red). The icl is more intensely labelled with Cav-1 than the ocl. The ICC are either c-kit- or cav-1-positive. **F**: double labelling with µSMA (red) and Mr2 (green). The icl is more intensely labelled with Mr2 than the ocl; both layers are αSMA-positive. **D–F**: Bar = 20 µm.

**Figure 2:**
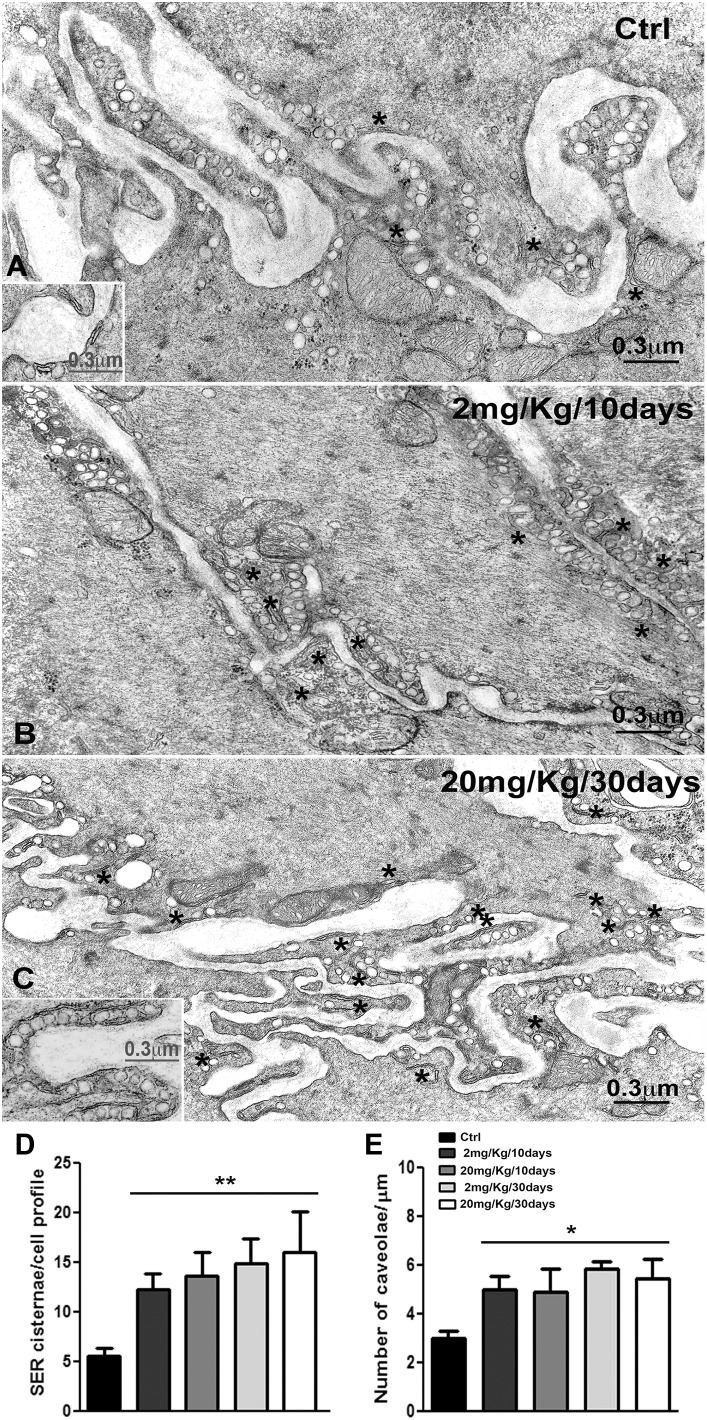
Transmission electron microscopy of the inner circular layer (icl). **A**: Controls. The profile of the cells of the icl is very irregular, with thin and interconnected ramifications, and the plasmalemma is rich in caveolae (asterisks). Several cisternae of the smooth endoplasmic reticulum are identifiable close to plasmalemma and caveolae. Inset: detail to evaluate the SER cisternae (asterisks) length and their relationship with the caveolae and the plasmalemma. **B**: Rat treated with OB at 2 mg/Kg for 10 days. Caveolae (asterisks) are particularly numerous, as well as the cisternae of the smooth endoplasmic reticulum. **C**: Rat treated with OB at 20 mg/Kg for 30 days. Caveolae are still more numerous than in controls and the cisternae of the smooth endoplasmic reticulum (asterisks) are long and closely apposed to the plasmalemma and the caveolae. Inset: detail to evaluate the length of the SER cisternae (asterisks), the richness in caveolae and their inter-relationship. **A–C and Insets**: Bar = 0.3 µm. **D**: quantification of the number of SER cisternae per cell profile shows a significant increase in all groups of treated rats compared to controls *p<0.05. **E**: quantification of the number of caveolae/µm demonstrates a significant increase in all group of treated rats compared to controls **p<0.01.

At the submucosal border of the CM (Fig. 1A) the ICC form an almost continuous layer closely apposed to the SMC of the CM inner portion (icl). The icl cells have an extremely irregular profile and many processes (Fig. 1A, B) are thinner (Fig. 1C) and richer in caveolae and cisternae of SER (figs. 1B, 2A) than those of the outer portion of the CM (ocl). The SER cisternae are close to plasmalemma and/or caveolae and their maximal length is 0.28 µm (range: 0.14–0.17 µm) (inset Fig. 2A). The cytoplasm is filled with thin filaments and mitochondria are distributed either near the nucleus or at cell periphery. Many nerve endings, some containing agranular synaptic vesicles and others granular synaptic vesicles, are close to the icl cells and intermingled with the ICC.

#### OB treated rats

At 10 days of OB treatment, with 2 mg/kg or 20 mg/kg, caveolae and SER cisternae are more numerous than in controls (Fig. 2A), especially in the icl cells (Fig. 2B). The SER cisternae, still close to the plasmalemma and the caveolae, are longer than in controls; their maximal length is 0.63 µm (range: 0.4–0.5 µm) and 0.52 µm (range 0.35–0.45 µm) in 2 and 20 mg/Kg treated rats, respectively. At 30 days, with both OB doses, caveolae are still numerous and the cisternae of SER are even more longer, especially in the cells of the icl (Fig. 2C), and their maximal length is 0.77 µm (range: 0.35–0.48 µm) and 0.8 µm (range: 0.3–0.45 µm) in 2 and 20 mg/kg treated rats, respectively (inset Fig. 2C). Statistical analysis demonstrated that the number of caveolae and of SER cisternae was significantly increased in all groups of treated animals compared to controls (Fig. 2D, E).

**Figure 3:**
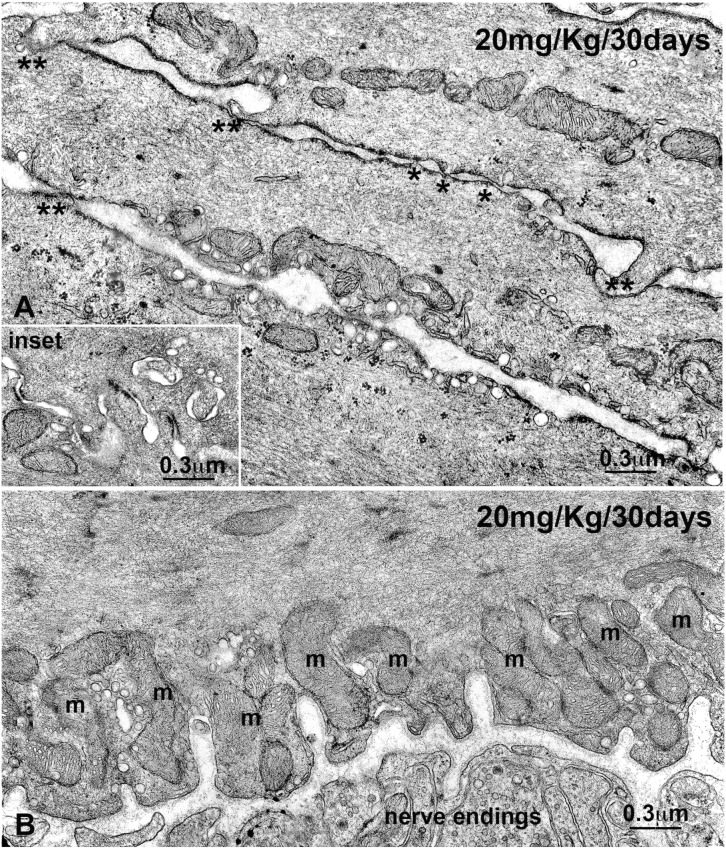
Transmission electron microscopy of the outer circular layer (ocl). Rats treated for 30 days with 20 mg/Kg of OB. **A** and **inset**: Numerous cell-to-cell specialized junctions (asterisks) connect the smooth muscle cells of the ocl to each other. **B**: Clusters of mitochondria (m) are aligned along the cell periphery. On the lower side, there is a nerve bundle with numerous nerve endings filled with synaptic vesicles. **A, B** and **inset**: Bar = 0.3 µm.

Noteworthy, at 30 days, the ocl cells show numerous specialized cell-to-cell contacts (Fig. 3A and inset Fig. 3A), and both the icl and ocl cells possess large clusters of mitochondria aligned along the cell periphery (Fig. 3B). In treated animals, no significant change in caveolae and SER richness is detected in the ICC, and in synaptic vesicles content and typology of the nerve endings (data not shown).

### Immunohistochemistry and western blot (WB)

#### Controls

**Figure 4:**
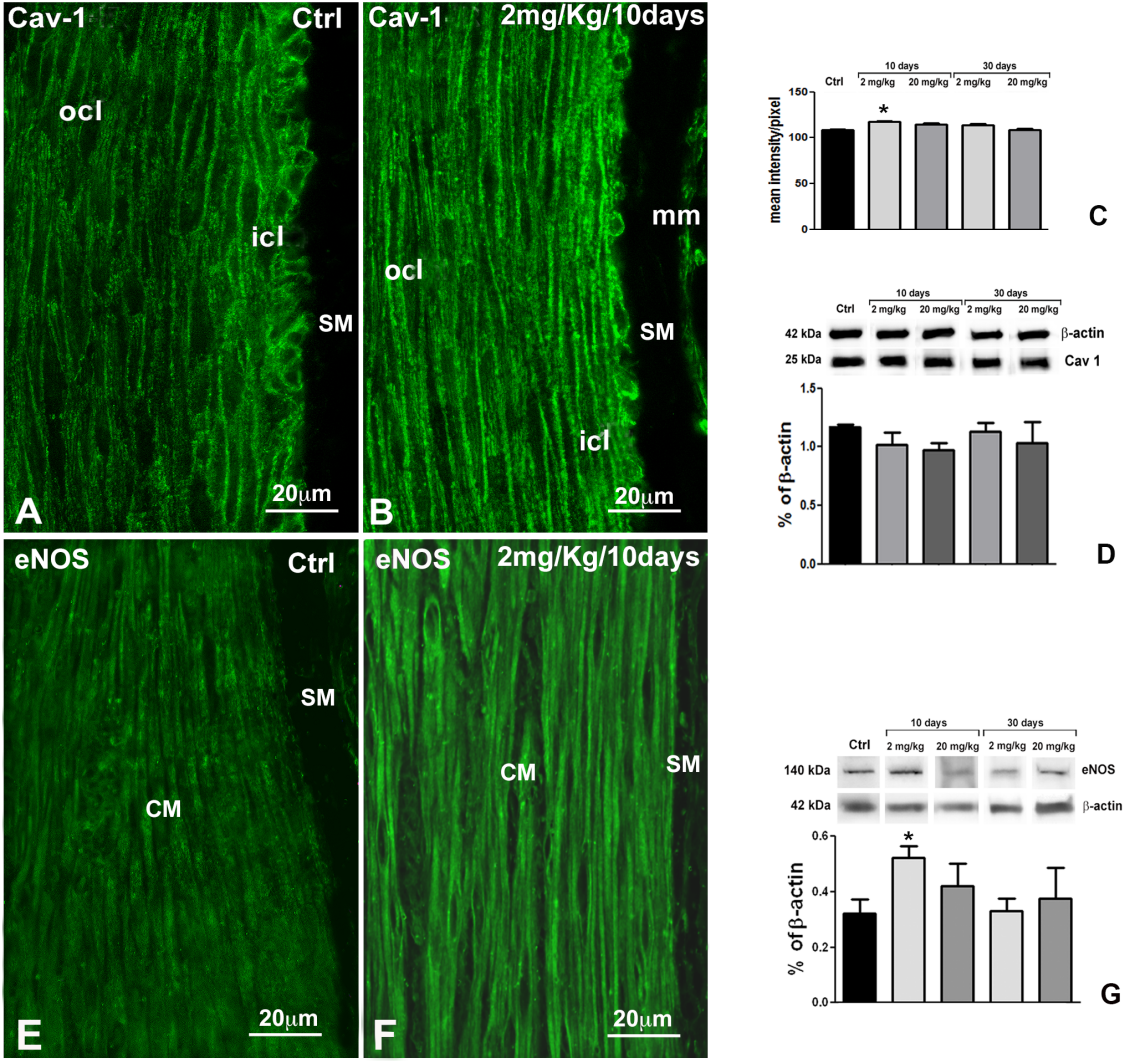
Immunohistochemistry (IHC) and western blots (WB) for Cav-1 and eNOS. **A**, **B**: IHC. Cav-1-immunoreactivity (IR) appears as small bars located along the plasma membrane of the cells both in the control group (**A**) and in the rats treated for 10 days (**B**). The Cav-1-IR in controls is more intense in the icl respect to the ocl cells. In the treated rats, Cav.1-IR intensity is increased in both layers. **C**: Quantification of Cav-1 intensity in the circular muscle layer. A significant increase in Cav-1 intensity is observed after 10 days of treatment (p<0.05). **D**: WB. Representative bands of Cav-1-IR and their quantification in control and treated rats. No significant change was seen. **E, F**: IHC. In controls (**E**), the eNOS-IR appears as granules mainly located at the periphery of the smooth muscle cells, with no differences between the icl and the ocl. In the rats treated for 10 days (**F**), the labelling intensity is increased in all the cells of the circular muscle (CM). SM: submucosa. **G**: WB. Representative bands of eNOS-IR and their quantification in control and treated rats. The increase at 10 days of OB treatment is significant (p<0.05) as compared to controls. **A, B, E, F**: Bar = 20 µm.

**Figure 5:**
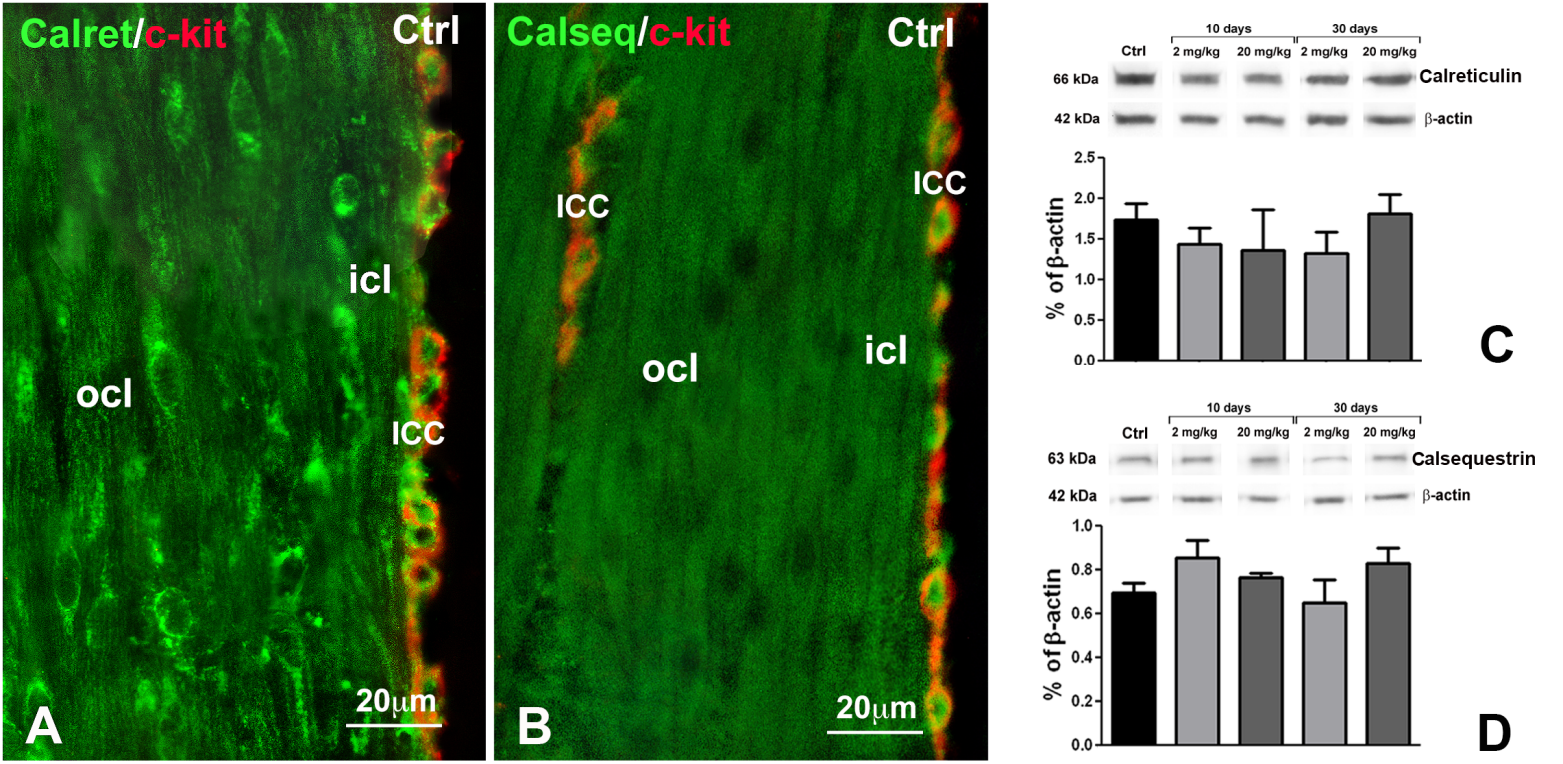
Immunohistochemistry (IHC) and western blots (WB) for Calret and Calseq. **A** and **B**: IHC. Controls. In (**A**), double labelling for Calret (green) and c-kit (red), in (**B**), double labelling for Calseq (green) and c-kit (red). The smooth muscle cells of both ocl and icl are positive, but no differences in the IR intensity are present between the two layers. The ICC are double labelled (green and red). Calret-IR appears as granules mainly distributed at cell periphery, while Calseq-IR is intracytoplasmatic. **A**, **B**: Bar = 20 µm. **C, D**: WB. Repre-sentative bands of Calret-IR (**C)** and Calseq-IR (**D**) and their quantification in control and treated rats. No significant change was seen at any time of duration of OB treatment for both markers.

**Figure 6:**
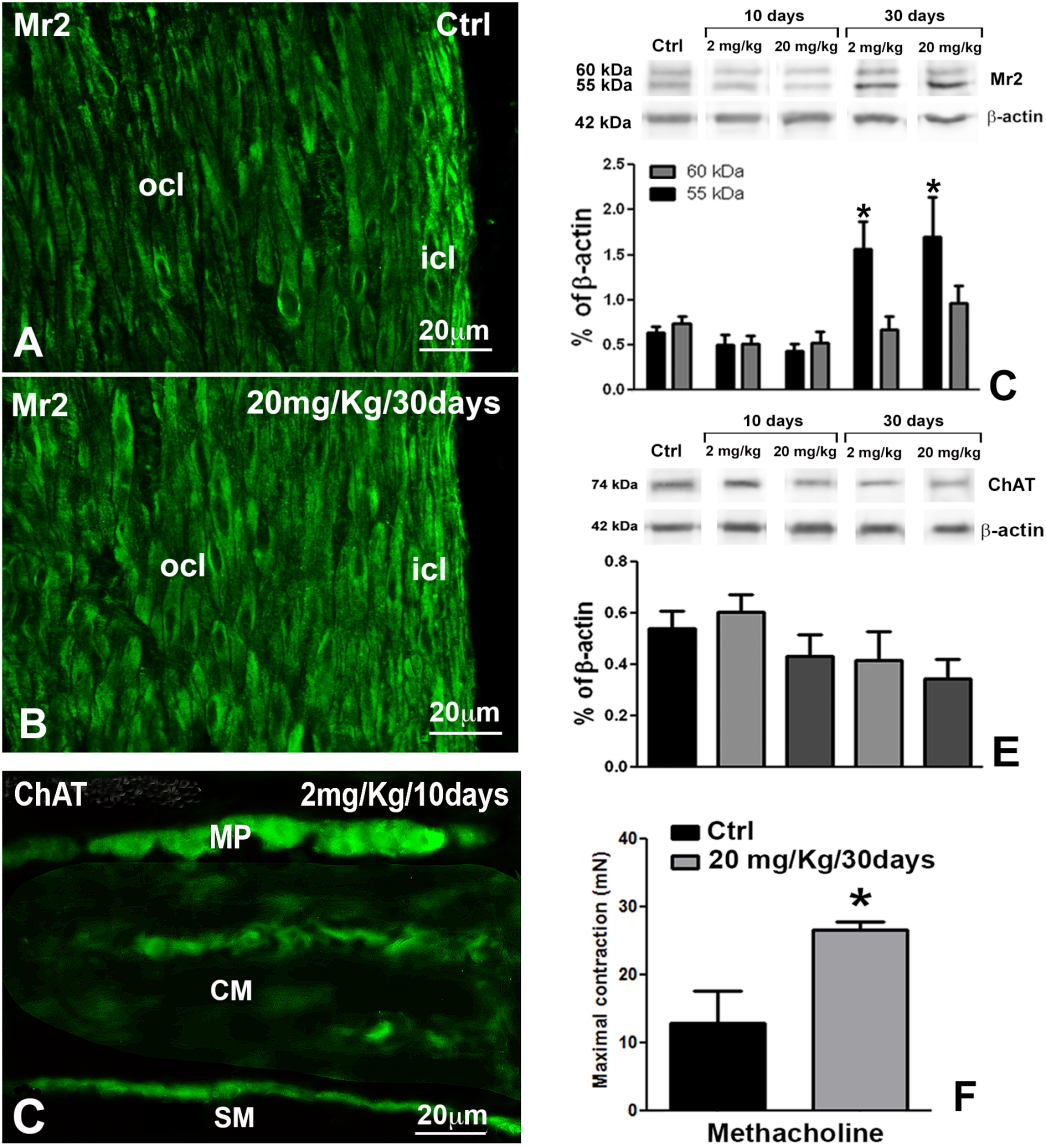
Immunohistochemistry (IHC) and western blots (WB) for Mr2 and ChAT and contractile effect of muscarinic agonist. In controls (**A**), Mr2-IR is granular, mainly distributed at cell periphery and more intense in the icl than in the ocl; in the animals treated for 30 days (**B**), the intensity of the IR is increased in the smooth muscle cells of both icl and ocl. **C**: As in the control, in the 10-day treated animals, there are ChAT-IR neurons at the myenteric plexus (MP) and nerve fibers within the circular muscle layer (CM), many of which are closely apposed to its submucosal border. SM: submucosa. **A–C**: Bar = 20 µm. **D**: WB analysis for Mr2 identifies two distinct bands with different MW. At 30 days of OB treatment, with both doses a significant increase in Mr2-IR is found mainly due to the band with the lighter MW. *p<0.01 versus controls and 10 day-treated animals. **E**: WB analysis of ChAT demonstrated that after 10 days of treatment there is an increase and after 30 days a decrease; however, none of these changes is significant. **F**: Contractile effect induced by a maximal concentration (1 µM) of the muscarinic agonist methacholine on circular muscle strips of proximal colon from control (black) and otilonium bromide (20 mg/kg/30 days) pre-treated (grey) rats. Each column is the mean ± s.e.m. of 6 experiments.

The two subdivisions of the CM are clearly distinguishable by immunolabelling for their size and shape and for some differences in immunoreactivity (IR). Indeed, α-smooth muscle actin-IR (αSMA-IR) (Fig. 1D), Caveolin-1-IR (Cav-1-IR, figs 1E, 4A) and muscarinic receptor2-IR (Mr2-IR, figs. 1F) are more intense in the icl cells than in those of the ocl. Conversely, endothelial nitric oxide synthase-IR (eNOS-IR, Fig. 4D), calreticulin-IR (calret-IR, Fig. 5A) and calsequestrin-IR (calseq-IR, Fig. 5B) show the same intensity in both the icl and ocl cells. To note, the labelling of αSMA, eNOS and calseq is located in the cytoplasm (figs.1D, F, 4D, 5B), that of calret and Mr2 is mainly distributed along the cell periphery (figs. 5A, 6A) and that of Cav-1 is definitively located along the plasmalemma (figs. 1E, 4A).

The ICC are c-kit-, cav-1-, calret-, calseq- (figs. 1D–E, 5A, B) and eNOS- (data not shown) positive, but are αSMA- (Fig. 1D) and Mr2-negative (data not shown). Moreover, calseq- and calret-IR are present also in neurons of the enteric plexuses (data not shown).

#### OB treated rats

Immunoreactivitiy of several of the molecules tested in the icl and ocl cells show changes in their intensity according to the time and dose of OB exposure.

*Molecules involved in calcium handling and storage.* At 10 days of OB treatment, Cav-1-IR and eNOS-IR intensities are increased (Fig. 4B, F) in comparison to controls (Fig. 4A, E), in both CM layers. Quantitative analyses of Cav-1-IR (Fig. 4C) and eNOS-IR (data not shown), reveals a significant increase (p<0.05) in treated rats compared to controls. WB analysis for Cav-1 does not display any difference in the protein expression between control and treated animals (Fig. 4D), while WB analysis for eNOS demonstrates an increase that reaches the significance at 2 mg/kg only (p<0.05) (Fig. 4G). At 30 days of OB treatment, immunohistochemistry (data not shown) and WB analyses (Fig. 4D, G) do not show any change in Cav-1 and eNOS expression between controls and treated rats. To note, calret-IR and calseq-IR, either by immunohistochemistry (data not shown) or WB analysis (Fig. 5C, D), do not show any significant change after OB treatment.

*Molecules involved in excitatory neurotransmission.* At 10 days of OB treatment, no significant change in Mr2- and ChAT-IR is detected by immunohistochemistry and WB analysis (Fig. 6D, E). To note, WB analysis for Mr2 identifies two distinct bands with different MW. At 30 days of OB treatment, a significant increase (p<0.05) in Mr2-IR is found, either by immunohistochemistry (Fig. 6B) or WB analysis (Fig. 6D). Interestingly, the WB increase is mainly due to the band with the lighter MW. At 30 days, a small decrease in ChAT content is observed (Fig. 6E).

### Functional experiments

At the end of the stabilization period, the colonic strips from control and treated rats show a similar resting tension (2.57±0.36 mN (n = 6) and 3.48±0.42 mN (n = 6) for controls and treated rats (20 mg/kg/day for 30 days), respectively (p>0.05). The Mr2 agonist methacholine induces a tonic contraction of the circular muscle of the proximal colon in controls and OB treated rats. In the rats treated with 20 mg/kg for 30 days, the contraction induced by methacholine is significantly (p<0.05) increased compared to controls, the mean values being 12.9±2.1 and 26.6±0.5 mN, respectively (n = 6 each group) (Fig. 6F).

## Discussion

The present findings demonstrate that in control animals the inner portion of the CM shows a higher expression of several markers compared to the outer portion, and is richer in organelles involved in calcium handling and storage. After chronic OB treatment, both CM portions undergo similar ultrastructural and immunohistochemical changes, but with a different time course. An increase in calcium-related organelles and in Cav-1 and e-NOS content is observed after 10 days of treatment. A significant increase in Mr2 expression and in CM contractility after exposure to a cholinergic agonist is detected in 30 days treated rats.

### The inner circular layer (icl)

Ultrastructural studies have already demonstrated that the CM of the rat proximal colon is subdivided in an inner (icl) and outer (ocl) portion differing in morphology and size of their SMC [1]–[3]. However, to date no information was available on possible differences in the expression of specific markers between the two portions. The present data showed that the SMC of the icl are particularly thin, possess several branches by which they contact each other forming a dense network, and are richer in SER, caveolae, Cav-1, αSMA and Mr2 than the SMC of the ocl. The intense Cav-1-IR is likely related to the richness in caveolae. The intense αSMA-IR might depend on the high content in the thin contractile filaments that fill the icl cell cytoplasm. The intense Mr2-IR likely indicates the icl cells are a preferred target of the neighbouring nerve endings, many of which are ChAT-IR (Fig. 6D). Conversely, the expression of calret, calseq and eNOS is similar between the icl and ocl cells. Reasonably, all these features should reflect a peculiar role of the icl in colonic contractility. Presently, we do not have enough data to identify this role and we can only make some hypotheses. The contacts with the submucosal ICC, i.e. the colonic pacemaker cells, and the richness in Mr2 make the icl cells the first target of the ICC electrical activity and excitatory inputs, respectively. When one or both of these inputs trigger the icl, it contracts forming a tight ring able to favour, from one side, the water and electrolyte absorption, and from the other side, together with the outer portion, the progression of the semisolid content present in the proximal colon.

### Structures and molecules involved in calcium storage and handling

In the rats chronically treated with OB a consistent increase in caveolae and SER, already appreciable at 10 days, was seen in the CM, especially in the icl. Moreover, a peculiar peripheral distribution of mitochondria in all the CM cells and an increase in specialized cell-to-cell contacts in the ocl were present following 30 days of OB treatment.

Immunohistochemistry and/or WB showed a significant change in the expression of eNOS and Cav-1 while the other molecules tested were unchanged. eNOS expression in the SMC has been previously demonstrated and it has been hypothesized that this enzyme is bound to the mitochondria handling the calcium storage and release from these organelles to favour muscular relaxation [16], [17]. Noticeably at 10 days, eNOS expression was markedly increased. The apparent discrepancy between the reduced calcium availability and the increase in the calcium-dependent eNOS can be explained. It has been reported that low levels of NO represent a stimulus for constitutive NOS transcription and synthesis [16], [18] and that the SMC respond to similar stimuli very rapidly [19]. In this regard, we previously demonstrated that chronic OB exposure induced a significant increase in the muscular nNOS at 10 days [12]. Therefore, this early increase in eNOS might represent the attempt by the SMC to counteract the decrease in intracellular calcium availability due to the OB block of calcium entry [11], [20], [21]. The return to the eNOS level similar to controls at a longer time of OB exposition (30 days) might be the consequence of a sort of adaptation by the cell to the lower level of intracellular calcium.

A modest, although significant, increase in Cav-1 intensity was present at 10 days of OB treatment, in agreement with the higher richness in caveolae seen under TEM. Cav-1 is a structural protein linked to the membrane of the caveolae and associated with the L-type Ca^2+^ channel [22], one of the main targets of OB [11], [20], [21]. We have previously demonstrated that chronic treatment with OB provokes a redistribution of the L-type Ca^2+^ channels in the cytoplasm instead of only on the plasmalemma. This redistribution likely impairs the possibility of association between Cav-1 and L-type Ca^2+^ channels and, consequently, in an attempt to favour this association the cell increases the number of caveolae. This increase is probably present from the first days of OB treatment and it remains at least up to 30 days. The discrepancy between immunohistochemical and WB data that showed no increase in Cav-1 content could be due to the fact that WB was performed on fully thick colonic samples comprehensive of the entire muscle coat plus submucosa and mucosa.

No change in calret and calseq expression was seen at any dose or at any time during the course of OB treatment in spite of the increased extension of SER observed under TEM by 10 up to 30 days. According to literature, calret and calseq intracellular distribution is not limited to the SER but is extended to other organelles [23]. Indeed, in our hands, the two antibodies labelled not only the SMC and the ICC but also the neurons, cells poor in SER. Therefore, the absence of any association between calret- and calseq-IR and SER extension is not surprising. Also, we cannot exclude that small changes in these marker expressions are present in the OB treated rats, but they could be under the threshold of our revelation system.

### Cholinergic system

In the treated animals, Mr2-IR was significantly increased after 30 days. The OB properties to block the Mr2 receptors are well known [10], [21]. Therefore, it is reasonable to hypothesise that the persistence of the drug induces the cell to counteract the OB block increasing the receptor expression. In this regard, the functional results showing a significant increase in muscle contractility to the muscarinic agonist methacholine compared to controls seem to indicate a presence of cholinergic hyper-responsiveness. As confirmation, electrophysiological studies showed that excitatory JP was significantly larger in treated animals as compared to controls [13]. The data we presently obtained at 30 days of OB treatment, such as the numerous mitochondria aligned close to the plasmalemma, many caveolae, extended SER, numerous and specialized cell-to-cell junctions, might be interpreted as signs of SMC adaptation to the presence of the drug aimed at maintaining its contractile activity.

### Interstitial cells of Cajal (ICC)

No ultrastructural or immunohistochemical change was observed in the ICC after OB treatment, although these cells, similarly to the SMC, are rich in caveolae, SER, mitochondria and express several of the SMC markers, such as Cav-1 [22], [24], eNOS, [17], calret and calseq [22] and present data. A possible explanation for these results likely relies on the absence of L-type Ca^2+^ channels and Mr2 in the ICC. The findings that the amplitude and frequency of slow waves [13] and the spontaneous motility [12] were not modified by OB treatment further underlie the ICC are not involved in the drug action.

## Conclusions

The present findings provide new information on immunohistochemical peculiarities of the icl that are in favour of a specific role of this portion in the colonic motility, a role that deserves to be elucidated in further studies. After chronic OB treatment, both inner and outer CM portions undergo significant ultrastructural and immunohistochemical changes, thus confirming that the CM is a preferred OB target.

Several studies have led to the conclusion that the OB block of the L-type Ca^2+^ channels constitutes a significant portion of its therapeutic effects [11], [12], [19]. However, the inhibition of excitatory neurotransmission [25], due to OB antagonism on neurokinin and muscarinic receptors [10], [21], is also considered important. In regard to the neurokinin-mediated neurotransmission, we recently demonstrated that chronic OB treatment modifies the expression and the function of the NK1receptors, but not of NK2 ones. However, this effect was considered indirect, due to the prolonged block of the L-type Ca^2+^ channels induced by the drug. Therefore, on the basis of the previous and present findings, we hypothesise that OB, when chronically administered, directly interacts with two targets, the L-type Ca^2+^ channels and the muscarinic receptors.

Intriguingly, the detection of significant ultrastructural changes in the SMC of the OB treated rats allows speculating that the effects of this drug might be long lasting, even following its interruption. In our experimental conditions we cannot evaluate whether these changes are beneficial or not, but we can reasonably hypothesise that the SMC adapt well to the lower level of intracellular calcium.
